# Optimized Wound Healing Assay to Study Extracellular Vesicle-Driven Glioblastoma Cell Migration

**DOI:** 10.3390/mps9030081

**Published:** 2026-05-31

**Authors:** Concetta D’Antonio, Francesca Mantile, Gabriella Pocsfalvi, Giovanna L. Liguori

**Affiliations:** 1 Institute of Genetics and Biophysics (IGB), National Research Council of Italy, 80131 Naples, Italy; 2 Institute of Biosciences and Bioresources (IBBR), National Research Council of Italy, 80131 Naples, Italy

**Keywords:** extracellular vesicles, glioblastoma, cancer cell migration, wound healing assay, silicone culture inserts, Mitomycin C, continuous exposure, quality assurance, critical process parameters

## Abstract

Cell migration is a fundamental process in cancer progression, playing a central role in tumor invasion and metastasis. This highly coordinated behavior is regulated by dynamic interactions between cancer cells and extracellular environment. Among the different tumor types, glioblastoma (GB) represents a particularly aggressive form of cancer in which enhanced migratory capacity is a key determinant of diffuse brain infiltration, tumor recurrence, and poor prognosis. In this context, extracellular vesicles (EVs) have emerged as important mediators, regulating cell migration in several cancer types, including GB. EVs are lipid bilayer-enclosed nano- and micro-sized particles, containing various bioactive molecules that can target specific recipient cells, thereby modulating cellular properties, including the migratory behavior. Among the available methods for studying cell migration, the wound healing assay is the most widely used. Although simple, cost-effective and not requiring sophisticated equipment, its reliability and reproducibility can be affected by technical variability and the diversity of existing protocols. Here, we present an optimized protocol for executing and analyzing a cellular wound healing assay designed to assess EV-mediated migration in GB cells. The protocol incorporates the use of silicone culture inserts to enhance wound homogeneity and reproducibility, together with continuous Mitomycin C incubation to inhibit cell proliferation without inducing cytotoxicity, enabling specific assessment of cell migration. We outline a step-by-step description of the procedure, detailing all required materials and equipment and highlighting critical steps, checkpoints, and key parameters. This method provides a robust framework for reproducible wound healing assays to investigate EV effects on GB cell migration.

## 1. Introduction

Cell movement is essential for many critical biological activities, affecting individual cells during division or differentiation as well as larger tissue structures, whose proper morphogenesis is ensured by coordinated migration. Furthermore, cell movement is involved in numerous pathological processes, such as immune activation, tissue repair, and cancer invasion and metastasis [1,2]. Cell migration can occur as single-cell or collective movement and is governed by complex relationships between cells and their extracellular environment and/or surrounding cells. Biochemical and biophysical external stimulation drives a finely tuned and orchestrated series of membrane and cytoskeletal rearrangements. These result in changes in polarity and protrusion formation, culminating in directional cell movement [3].

Extracellular vesicles (EVs) are key components of the extracellular milieu, and play an important role in maintaining cellular homeostasis and mediating intercellular communication. The term EVs refers to lipid bilayer nanoparticles or microparticles released by the cells into the extracellular space. EVs can travel into the body fluids and recognize target cells specifically through interaction between surface molecules on the membrane [4,5,6,7]. Interaction between EVs and recipient cells occurs through various mechanisms, including direct membrane receptor activation, or internalization through endocytosis, macropinocytosis or phagocytosis, with subsequent modulation of signaling pathways and gene expression [8,9]. EVs carry bioactive molecules such as proteins, nucleic acids, lipids, and metabolites, which reflect the state and cellular content of the cells of origin, and induce a variety of phenotypic response in target cells. For this reason, EVs are increasingly acknowledged as vital mediators that coordinate intercellular communication in both health and disease [10,11,12,13], including cardiac, metabolic and renal pathologies [14,15], as well as neurological disorders [16,17,18] and cancers [4,19,20]. EVs are key drivers of cancer progression and metastasis. They modulate tumor cell traits such as proliferation, epithelial–mesenchymal transition, migration, invasion, dormancy, and chemoresistance, while shaping interactions with the tumor microenvironment and immune system [4,21,22,23,24,25,26,27]. Furthermore, EVs can be obtained from various donor cell types and applied to different recipient cells in translational research. Thanks to their intrinsic biocompatibility, low immunogenicity, and specific bioactivity, exogenous EVs, whether pristine or engineered for drug delivery, are poised to become the next-generation platforms for precision therapy, including in oncological applications [28,29]. In this context, tumor-derived EVs have attracted considerable attention as cancer-targeted therapeutic agents, both in immunotherapy and as drug delivery systems capable of efficiently transporting cargos to malignant cells [22]. Glioblastoma (GB) is an aggressive brain tumor with a poor prognosis, largely due to the highly infiltrative nature of GB cells, which hampers complete surgical removal and increases the likelihood of relapse. EVs are key players in regulating GB pathological features, including cell invasiveness and migration [30,31,32]. Therefore, identifying an EV-based therapy that can target the aggressive migratory behavior and invasiveness of GB cells is desirable.

Traditionally, two-dimensional (2D) GB monolayer cell cultures have been used for the rapid and easy testing of candidate anti-migratory drugs [33]. Various experimental methods have been developed to analyze and quantify cell migration features such as velocity, cell trajectory, and directionality. These include the traditional wound healing assay, the Boyden chamber, and the Zigmond chemotaxis chamber, as well as live cell imaging and single-cell tracking. More recent methods rely on transient agarose spots or three-dimensional (3D) microfluidic channels [34,35,36]; however, these approaches have not yet been widely adopted. The wound healing assay remains one of the most frequently used methods for studying cell migration [37]. This involves creating a cell-free area, or wound, in a cell monolayer, and then studying how the wound closes over time by capturing and analyzing images at different time points. The velocity of wound closure indicates how rapidly cells migrate into the wound. This assay is commonly used to compare the migration of different cell types or the effects of different treatments, including EV administration, on the migration of specific cells. Despite its widespread use, several articles highlight an increasing number of factors that can severely impact the reliability and reproducibility of the assay, if they are not adequately controlled [38,39,40,41,42], with protocols involving EVs and GB cells being particularly poorly validated.

Here, we present an optimized protocol for setting up, conducting, and analyzing a wound healing assay to study the influence of EVs on GB cell migration. The protocol uses silicone culture inserts to enhance the reproducibility of the wound and Mitomycin C continuous incubation to efficiently inhibit cell proliferation. The core methodology was first established in our previous study on the effect of NTERA2 teratocarcinoma-derived EVs on the U87 GB cell migration [43] and subsequently formalized in a standard operating procedure (SOP) for an insert-based wound healing assay [44]. The protocol presented here has been further refined to ensure higher reproducibility and detailed quantification. NTERA2 teratocarcinoma cells closely resemble neural stem/progenitor cells, as they are capable of differentiating into glia and neurons, and of locating and targeting glioma neoplasms in mouse models, particularly following treatment with retinoic acid [45,46,47,48]. Furthermore, it has been found that conditioned medium from NTERA2 cells affects GB cell behavior [47], suggesting that GB cells are responsive to signals emitted by NTERA2 cells.

Against this biological background, this article outlines the steps of the protocol, detailing the necessary materials and instruments, critical process parameters and potential issues and solutions. This formalized protocol provides a valuable tool to support the reproducibility of the experiments, while ensuring quality assurance of procedures and quality control of data.

## 2. Experimental Design

### 2.1. Preparatory Phase

#### 2.1.1. EV Samples

EV isolation and characterization were performed according to MISEV 2018 and 2023 [49,50], as previously reported [43]. Briefly, EV fractions obtained at 10,000 and 118,000× *g*, corresponding to medium/large and small EV-enriched fractions respectively, were isolated from NTERA2 cell culture medium using differential centrifugation. The identity of the EVs was confirmed by showing they possessed specific positive markers, such as the CD63 membrane-bound marker and the HSP70 luminal marker, while lacking negative markers specific of intracellular compartments (Calnexin) [43]. Cryo-electron microscopy (Thermo Fisher Scientific, Waltham, MA, USA) confirmed the physical structure of both the medium/large and small EV fractions, highlighting their delimitation by a lipid bilayer. The protein content of NTERA2 EVs was determined using the Micro BCA Protein Assay Kit (Thermo Fisher Scientific, Waltham, MA, USA; Cat. no. 23235), followed by a NanoDrop 1000 Spectrophotometer (Thermo Fisher Scientific, Waltham, MA, USA) measurement, whereas the size and concentration of nanoparticles was determined using Nanoparticle Tracking Analysis (NanoSight NS300; Malvern Panalytical, Malvern, UK) [43].

**Figure 1:**
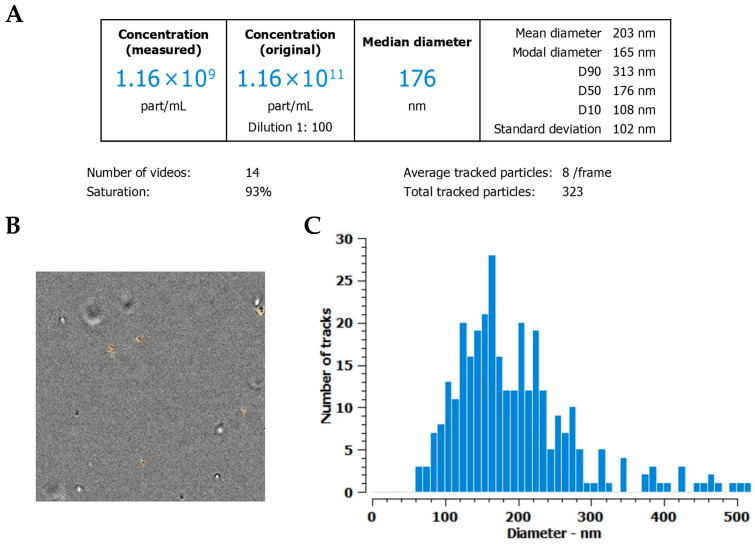
Example of interferometric light microscopy (ILM, Videodrop) analysis of NTERA2 extracellular vesicles. Shown are (**A**) ILM Videodrop output with particle concentration (particles/mL) and median diameter values, (**B**) a representative videomicrograph of the particles in real time, and (**C**) the corresponding size distribution profile. Particle size distribution shows that most particles range between 108 and 313 nm, with characteristic percentile values of D10 = 108 nm, D50 = 176 nm (median diameter), and D90 = 313 nm, indicating that 10%, 50%, and 90% of particles are below these respective diameters.

We previously investigated, and here further examine, the effects of NTERA2-derived EV fractions (isolated at 10,000× *g* for 30 min) on GB cell migration. In this study, all EV preparations refer specifically to fractions obtained at this centrifugation force. The protein content of each EV preparation was determined using the Micro BCA Protein Assay Kit in accordance with the manufacturer’s instructions. The absorbances of the reactions were measured at a wavelength of 562 nm, employing the NanoPhotometer NP80 (Implen GmbH, Munich, Germany). Interferometric light microscopy (ILM) analysis [51] was also performed on random batches to control the number and size distribution of the particles. We used the Videodrop instrument (Myriade, Paris, France), according to the manufacturer’s recommendations [52]. EV samples were diluted in PBS (filtered by 0.22 μm) to reach the optimal concentration range for ILM detection (typically 1:100). For each measurement, 7 µL of the diluted EV suspension was gently loaded into the Videodrop slide and allowed to equilibrate for a few seconds before acquisition. The Videodrop system detects nanoparticles in solution by analyzing the interference pattern generated by light scattered by each particle, enabling real-time tracking of their Brownian motion. Particle size distribution (median diameter, nm) and particle concentration (particles/mL) were quantified automatically using Videodrop’s integrated software (version qvir: 2.10.3.8978). The software also provided the mean, modal, and standard deviation of the hydrodynamic diameter for each acquisition. For each acquisition, a minimum of 300 particles were tracked to ensure robust estimation of particle parameters. For each EV batch, three to five technical measurements were acquired, and values were averaged to obtain the final particle parameters. This approach provides a rapid, label-free assessment of EV concentration and size distribution, with high reproducibility and without requiring sample fixation or additional processing. The results are shown in Figure 1 and Table 1.

The first two EV batches derived from independent cell preparations, while the third batch was a pooled reference preparation (not including the first two batches). The three batches showed an average particle size of 176.19 ± 7.37 nm and a particle-to-protein ratio of 1.92 × 10^8^ ± 4.35 × 10^7^ particles/μg, indicating uniformity and consistency across preparations. The ratio of our EV batches aligns with the ratio reported for large EV fractions of a similar size isolated from breast cancer cell lines, either after 16,500× *g* sedimentation alone or followed by density cushion centrifugation [53]. After quantification, EV batches were aliquoted and stored at −20 °C or −80 °C until the wound healing assay.

#### 2.1.2. Preparation of the Cells

For our assay, we used U87 GB cells from Merck (Rahway, NJ, USA; Cat. no. 89081402). The cells were grown at 37 °C in a humidified atmosphere containing 5% CO_2_, in Dulbecco’s Modified Eagle’s Medium (DMEM), high glucose and pyruvate (Gibco Invitrogen, Carlsbad, CA, USA; Cat. no. 11995065) supplemented with 10% (*v*/*v*) Fetal Bovine Serum (FBS, Gibco Invitrogen, Carlsbad, CA, USA, Cat. no. A5256701), and a penicillin–streptomycin solution (100 U/mL penicillin and 100 μg/mL streptomycin), from Gibco Invitrogen (Carlsbad, CA, USA; Cat. no. 15140122). Cells were maintained at low passage number (<3 passages post-thawing) for all assays. The cells were sub-cultured approximately every 3 days after thawing. For subculture, the cells were detached by incubation with Trypsin 0.25% (Gibco Invitrogen, Carlsbad, CA, USA; Cat. no. 15090046) at 37 °C in a humidified atmosphere (5% CO_2_) for 2–3 min. As Mycoplasma is an invisible contaminant that could alter the structure, metabolism and behavior of the cells [54], thereby affecting the reproducibility of results, we verified its absence in the cell cultures using the Mycoplasma PCR detection kit (Applied Biological Materials Inc., Richmond, BC, Canada; Cat. no. G238).

### 2.2. Wound Healing Assay

**Figure 2:**
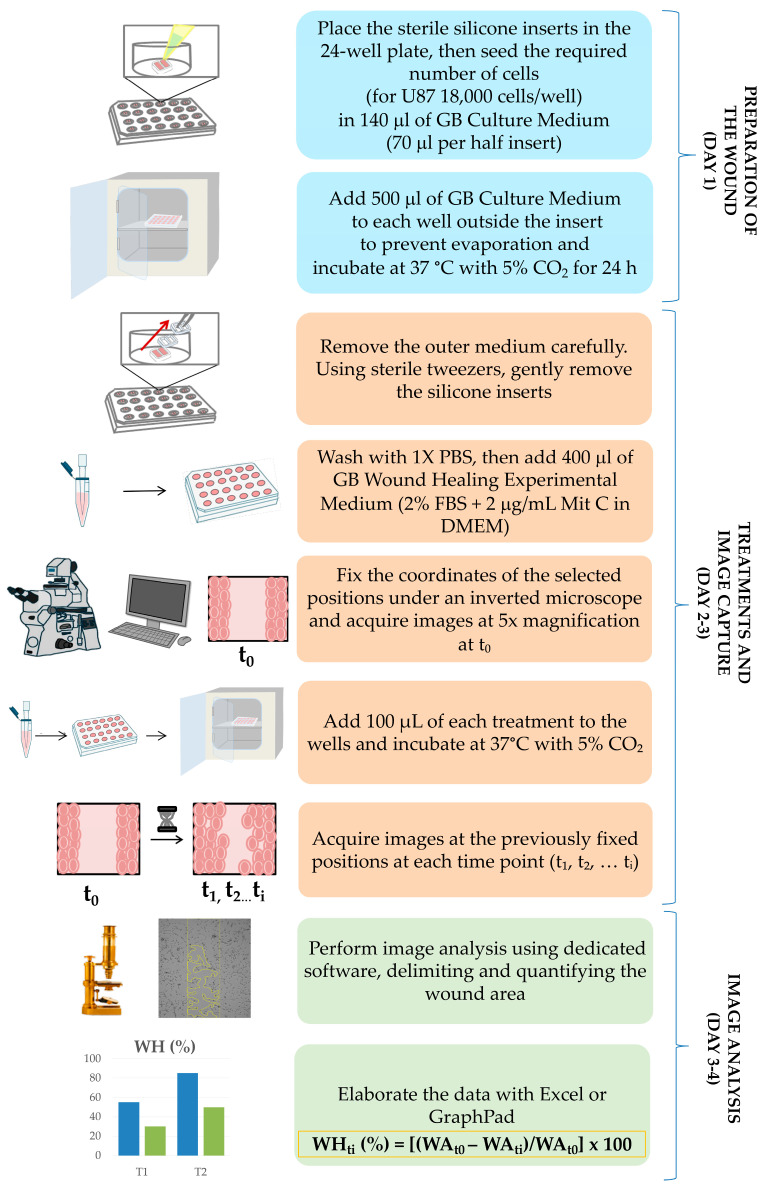
Experimental design of the wound healing assay, highlighting the main steps of the procedure. Abbreviations: CO_2_, Carbon Dioxide; DMEM, Dulbecco’s Modified Eagle’s Medium; FBS, Fetal Bovine Serum; GB, glioblastoma; Mit C, Mitomycin C; PBS Phosphate-Buffered Saline; WA, wound area; WH, wound healing.

The experimental design with the main steps of the protocol is shown in Figure 2.

Depending on the time of observation, the protocol takes 3–4 days to complete, and comprises three main phases:

1. Preparing the wound (Day 1);
2. Treatment and image acquisition at different time points (Days 2–3);
3. Image analysis (Days 3–4).

We recommend performing the assay for at least 24 h and no more than 42 h to preserve the healthy state of the cells. Control samples of untreated cells should also be included.

#### 2.2.1. Materials

##### Plasticware

- Tissue culture-treated culture dishes D × H 100 mm × 20 mm (Corning, New York, NY, USA; Cat. no. 430167);
- Stripette 2 mL (Cat. no. 4486), 5 mL (Cat. no. 4487), 10 mL (Cat. no. 4488), and 25 mL (Cat. no. 4489) Serological Pipettes, polystyrene, individually wrapped (Corning, New York, NY, USA);
- Bulk-Packed 1–200 μL (Cat. no. 4845) and 100–1000 μL (Cat. no. 4846) Pipette Tips (Corning, New York, NY, USA). Autoclave before use;
- Primo^®^ boil-proof microcentrifuge tubes 1.5 mL (Euroclone, Pero, MI, Italy; Cat. no. ET3415). Autoclave before use;
- Primo^®^ EZ tubes 15 mL (Cat. no. ET5015B) and 50 mL (Cat. no. ET5050B) conical centrifuge tubes (Euroclone, Pero, MI, Italy);
- 24-well clear tissue culture-treated multiple well plates (Corning, New York, NY, USA; Cat. no. 3524);
- Culture-Inserts 2 Well for self-insertion (ibidi, Gräfelfing, Germany; Cat. no. 80209).

##### Reagents

- Dulbecco’s Modified Eagle’s Medium (DMEM), high glucose, pyruvate (Gibco Invitrogen, Carlsbad, CA, USA, Cat. no. 11995065);
- Fetal Bovine Serum (FBS, Gibco Invitrogen, Carlsbad, CA, USA; Cat. no. A5256701);
- Penicillin (10,000 U/mL)—Streptomycin (10,000 μg/mL) solution (Gibco Invitrogen, Carlsbad, CA, USA, Cat. no. 15140122);
- 1× Phosphate-Buffered Saline (PBS) without Calcium, Magnesium or Phenol Red (Euroclone, Pero, MI, Italy; Cat. no. ECB5004L);
- 2.5% Trypsin (Gibco Invitrogen, Carlsbad, CA, USA; Cat. no. 15090046)
- 0.4% Trypan Blue Stain (Gibco Invitrogen, Carlsbad, CA, USA; Cat. no. 15250061);
- 10 mg/mL Mitomycin C solution (Sigma-Aldrich, St. Louis, MO, USA; Cat. No. M5353)
- 95% Paraformaldehyde (PFA) powder (Sigma-Aldrich, St. Louis, MO, USA; Cat. No. 158127);
- Glycerol 99% (Chem-Lab, Zedelgem, Belgium; Cat. No CL00.0724).

##### Lab-Prepared Cell Culture Media and Solutions

- Trypsin solution (0.25%, freshly prepared by diluting 2.5% Trypsin stock solution in 1× PBS);
- GB Culture Medium (DMEM supplemented with 10% *v*/*v* FBS, 100 U/mL penicillin, and 100 µg/mL streptomycin). This medium should be freshly prepared and stored at 4 °C for up to 30 days;
- GB Wound Healing Experimental Medium (DMEM freshly supplemented with 2% *v*/*v* FBS, 100 U/mL penicillin, 100 µg/mL streptomycin, and 2 μg/mL Mitomycin C);
- Mitomycin C solution (100 μg/mL freshly prepared by diluting 10 mg/mL stock in 1× PBS). The solution is promptly diluted in GB Wound Healing Experimental Medium to the desired final concentration (usually 2 µg/mL);
- 20% (*w*/*v*) PFA stock solution (prepared under a chemical fume hood by dissolving PFA powder in 1× PBS with gentle heating to 60 °C and stirring until fully dissolved). The solution is cooled to room temperature, aliquoted, and stored at −20 °C. Immediately before use, an aliquot of the stock is diluted in 1× PBS to a final concentration of 4% (*w*/*v*) PFA for cell fixation.

#### 2.2.2. Equipment and Software

- Laminar flow hood Class II biosafety for cell culture;
- Water jacketed CO_2_ incubator for cell culture (ThermoFisher, Waltham, MA, USA; Forma Series II);
- Inverted light microscope equipped with a motorized stage (Leica, Wetzlar, Germany; DMI6000), a Leica DFC 480 RGB (Max. Res. 2560 × 1920) video camera, an OrcaR2 B/W (Max. Res. 1344 × 1024) video camera, a computer and the related software (LAS AF, version 4.6, Leica, Wetzlar, Germany);
- ImageJ software (version 1.54g, NIH, Bethesda, MD, USA);
- Microsoft Excel software (version 16.54, Microsoft Corporation, Redmond, WA, USA);
- Microsoft PowerPoint software (version 16.54, Microsoft Corporation, Redmond, WA, USA);
- Adobe Photoshop CS6 (version 13.0 x64, Adobe Inc., San Jose, CA, USA);
- Fume hood (if PFA fixation is to follow);
- Levo Plus Electronic pipette Filler (DLAB Scientific Co., Ltd., Beijing, China);
- P20, P200, P1000 Pipettes (Gilson, Madison, WI, USA);
- Bürker chamber (Waldemar Knittel Glasbearbeitungs GmbH, Braunschweig, Germany);
- Sterile tweezers.

## 3. Procedure

### 3.1. Preparation of the Wound (Day 1)

1. Incubate GB U87 cells in a 100 mm culture dish in GB Culture Medium at 37 °C in a humidified atmosphere containing 5% CO_2_ until the cells reach 80% confluence.
2. **CRITICAL STEP** Remove the GB Culture Medium and wash the cells with 2 mL of 1× PBS. Incubate the cells at 37 °C in a humidified atmosphere (5% CO_2_) with 2 mL of 0.25% Trypsin solution for 2–3 min, until the cells detach from the dish. Add an equal volume of GB Culture Medium to stop the trypsinization process and collect the detached cells in a 15 mL conical tube.
3. Centrifuge at 80–100× *g* for 5 min, preferably with gentle acceleration/deceleration, to collect cells. Discard the supernatant and resuspend the cells in 1–2 mL of GB Culture Medium.
4. Prepare a 1:10 (*v*/*v*) cell dilution in final 0.2% Trypan Blue vital dye (adjusted with 1× PBS), then count the cells using a Bürker counting chamber. Gently pipette to obtain a homogeneous mixture, and load 10 µL into the Bürker chamber. Observe the mixture under an inverted microscope and count the number of viable (unstained) cells in at least four squares. Calculate the total number of cells based on the average cell count, the dilution factor, the chamber conversion factor, and the sample volume.
5. Calculate the number of cells required for the wound healing assay using the following formula:
  TOT N cells = N cells/well × N treatments × N replicates for each treatment
  The number (N) of cells to be seeded in each well must be sufficient to form a confluent or subconfluent cell monolayer 24 h after seeding. For U87 GB cells, we used 18,000 cells per well.
  **Note**:
  It may be helpful to set up three or four additional wells in case some wells encounter difficulties during wound formation.
6. Transfer the required number of cells for the entire experiment to a separate 15 mL conical tube.
7. **OPTIONAL STEP** Dilute to an intermediate volume (V_count_) of 0.5 mL of GB Culture Medium. Resuspend well using serological pipettes, and then count the cells again using a Bürker chamber (making a 1:3 to 1:5 *v*/*v* cell dilution in final 0.2% Trypan Blue) to ensure the desired number of cells is present.
8. Add GB Culture Medium to obtain the required V_f_, according to the formula:
  V_f_ = 70 μL (V for half insert) × 2 × N wells
9. **CRITICAL STEP** Under a laminar flow hood, open the box of inserts and place one in the center of each well of a 24-well plate, using sterile tweezers. Make sure that the silicone insert is firmly attached to the plate and will not move.
10. **CRITICAL STEP** Always resuspend the cells just before aliquoting and add 70 μL of cell suspension to each side of the insert (we used 9000 cells for each insert side, and then 18,000 cells in 140 μL for each well). Add a gentle, central flow of the cell suspension, dispensing the suspension against the wall of the insert, to ensure a smooth and regular flow. Allow cells to settle undisturbed for 10–15 min.
11. Add 500 μL of GB Culture Medium to each well, outside the insert, to prevent evaporation. Incubate at 37 °C in a humidified atmosphere containing 5% CO_2_ for at least 24 h to allow the cells to reach the required confluence.

### 3.2. Treatment (Day 2)

1. **CRITICAL STEP** Check the cell density under a microscope and ensure a monolayer is visible. Then, remove the medium outside the insert.
**Note**
  The cell density should be sufficient to allow monolayer formation, but not so high as to promote sphere formation.
2. **CRITICAL STEP** Using sterile tweezers, gently pull one corner of the inserts away from the wells. Check under the microscope that the monolayer is still attached and that the wound is homogeneous.
**Note**
  Using the ibidi two-well culture-inserts, the generated wound has a width of 500 μm. Irregular wounds might affect the reproducibility of the assay.
3. **CRITICAL STEP** Wash the wells with 1× PBS and add 400 μL of GB Wound Healing Experimental Medium containing 2% FBS and 2 μg/mL of Mitomycin C to inhibit cell proliferation.
**Note**
  The concentration of Mitomycin C to be used in the Experimental Medium may differ for each cell line and should be determined in advance to inhibit cell proliferation without causing cytotoxicity.
4. **CRITICAL STEP** Place the multiwell under the motorized inverted light microscope. The coordinates of the selected positions within the wound area are identified using the microscope control software and stored using the stage-positioning system (e.g., as a .maf file in Leica LAS AF software). Maintain consistent focus, exposure, and imaging settings across all positions. Once the positions are recorded, acquire the initial images corresponding to the *t*_0_ time point. If a motorized stage is not available, images can be acquired manually at a consistent, predefined distance from the insert edge, using the field of view and a calibrated scale as references. For both acquisition modes, it is recommended to capture two images per well at 5× magnification to obtain a representative view of the wound. Avoid regions very close to the outer edge of the insert, which are more irregular, and ensure that images do not overlap. Assign clear and consistent filenames (e.g., including sample ID, well number, position, and time point) and save all images in TIFF format to preserve quality for downstream analysis.
5. Under the hood, add 100 μL of the EV sample diluted in GB Wound Healing Experimental Medium (for untreated samples, add 100 μL of the GB Wound Healing Experimental Medium alone) in triplicates. Prepare a single master mix of 300 µL, then distribute 100 µL per well for each triplicate. Add the EV suspension gently, to ensure a smooth and uniform flow and to avoid disturbing the cell monolayer. Incubate at 37 °C in a humidified atmosphere containing 5% CO_2_.
6. **CRITICAL STEP** At predefined time points (e.g., *t*_1_ = 16 h, *t*_2_ = 24 h, *t*_3_ = 42 h), acquire new images from the same previously defined positions used in earlier imaging sessions. For motorized stage systems, use the saved coordinates (e.g., from Leica LAS AF .maf files) to return to the original positions. For manual acquisition, use the predefined spatial references. In both cases, visually verify alignment before capturing images. Acquire images following the same settings and naming conventions as for *t*_0_ and transfer them from the microscope computer to the laboratory workstation for further storage and data analysis.
7. **OPTIONAL STEP A** At the end of the experiment, assess cell viability using Trypan Blue staining. Collect and store the conditioned medium at −20 °C for downstream analysis, and detach the cells by adding 200 µL of 0.25% Trypsin per well, following the instructions in Day 1, Step 2. Centrifuge the cell suspension at 100× *g* for 5 min, discard the supernatant, and resuspend the cell pellet in 200 µL of GB Culture Medium. Count the cells using a Bürker counting chamber, as described in Day 1, Step 4, at the appropriate dilution.
8. **OPTIONAL STEP B** Alternatively, after collecting and storing the conditioned medium at −20 °C, fix U87 cells with 4% PFA for 15–20 min. Wash twice with 1× PBS, and store in a PBS/glycerol (1:1, *v/v*) solution at −20 °C until downstream analyses (e.g., immunofluorescence).

### 3.3. Image Analysis (Day 3)

1. **CRITICAL STEP** Use image analysis software (e.g., ImageJ or CellProfiler) to define and quantify the wound area in different images taken at different times. You can define the area both manually or using the ImageJ Wound Healing Size Tool plugin, described by Suarez-Arnedo and coauthors [55]. Define the ideal wound area per image field in square millimeters (mm^2^), based on the type of insert, microscope magnification, and camera used to capture images. In our study, cell samples were excluded when the total wound area at *t*_0_, calculated across two imaging fields, deviated by more than 10% from the corresponding expected value.
2. Use Microsoft Excel and/or GraphPad Prism for data analysis and graphing, as well as Adobe Photoshop and Microsoft PowerPoint for image processing and figure assembly. Two main parameters can be calculated and plotted:

- **The Wound Healing** at different time points expressed as the percentage of healing compared to initial wound area:
  WH*_t_*_i_ (%) = [(WA*_t_*_0_ − WA*_t_*_i_)/WA*_t_*_0_] × 100
  WH*_t_*_i_ (%) is the percentage of wound healing at a given time *t*_i_;
  WA*_t_*_0_ is the area of the wound at time *t*_0_;
  WA*_t_*_i_ is the area of the wound at a given time *t*_i_.
  Quantify wound closure based on the total gap, calculated by summing the area of both imaging fields to minimize potential bias caused by spatial heterogeneity.
- **The Absolute Rate of Gap Closure** (in mm^2^ over time) calculated as the slope of the line obtained by plotting the area of the wound covered by cells (*y*-axis) against time (*x*-axis), before reaching the saturation phase.

The assay should be repeated at least three times using different cell batches at similar passage numbers, and different EV batches. Statistical analysis (including Student’s *t*-test, one-way ANOVA and Tukey’s HSD test) is essential for assessing the reproducibility and significance of the data.

## 4. Results

**Figure 3:**
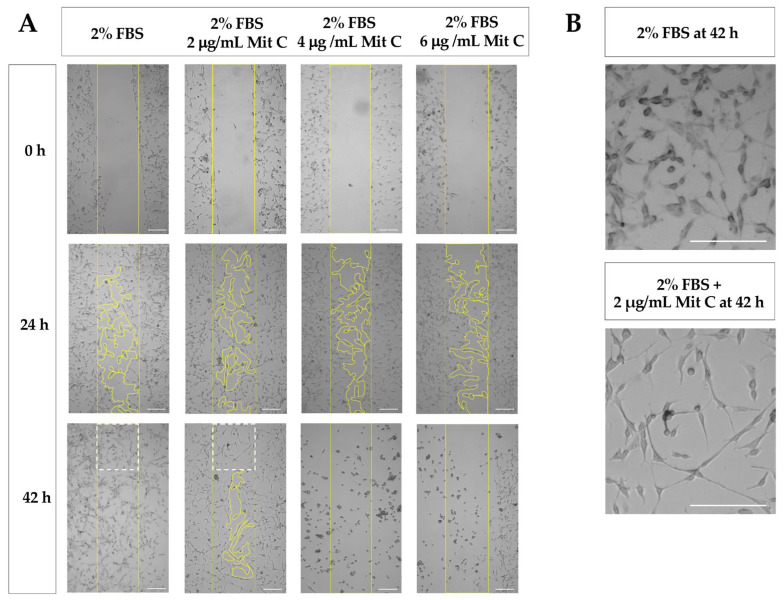
Time-dependent wound healing of U87 cells at different Mitomycin C concentrations. (**A**) Representative images at 0, 24, and 42 h after wounding. Thick yellow lines indicate the cell-free area at each time point; thinner yellow outlines at 24 and 42 h show the original wound area at 0 h. (**B**) Magnified views of dotted areas in (**A**) for 2% FBS ± Mitomycin C at 42 h. All scale bars represent 250 µm. Abbreviations: FBS, Fetal Bovine Serum; Mit C, Mitomycin C.

**Figure 4:**
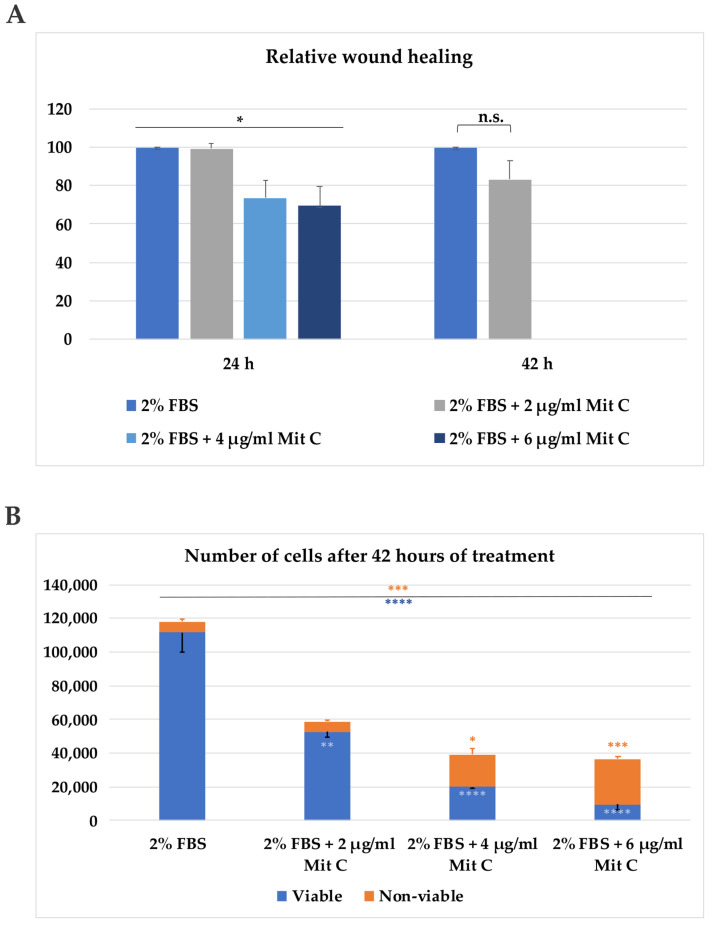
Mitomycin C dose-dependent effects on U87 wound healing and cell number. (**A**) Wound closure quantified as percentage relative to cells in 2% FBS without Mitomycin C. Data are presented as the mean ± standard error of the mean (SEM) from three independent experiments (each performed in triplicate). Statistical analysis was performed using one-way ANOVA followed by Tukey’s test at 24 h, and *t*-test comparisons at 42 h. (**B**) Number of viable (blue) and non-viable (orange) cells at the end of the treatment (42 h). Data represent the mean ± SEM from the same three independent experiments, each performed in triplicate. Lower dark bars refer to viable cells and upper orange bars refer to non-viable cells. Statistical analysis was performed using one-way ANOVA followed by Tukey’s test, with blue asterisks referring to viable cells and orange asterisks to non-viable cells. For each group, statistical significance is reported as n.s. (not significant), * (*p* < 0.05), ** (*p* < 0.01), *** (*p* < 0.001), and **** (*p* < 0.0001), compared to the 2% FBS condition without Mitomycin C.

To ensure high reproducibility, particular emphasis was placed on critical experimental parameters. Specifically, the determination of the optimal Mitomycin C concentration was investigated, while the handling of silicone inserts and the quantification analysis of the resulting wound area were refined to minimize experimental variability. Figure 3 and Figure 4 compare the effects of different Mitomycin C concentrations (2, 4, and 6 μg/mL) on cell motility and viability. Table 2 shows the wound areas and percentage of wound healing for each image.

At 24 h, ANOVA revealed a significant overall difference among the four tested conditions (2% FBS without Mitomycin C and Mitomycin C, at concentrations of 2, 4, and 6 µg/mL). However, post hoc pairwise comparisons did not detect any statistically significant difference between individual groups (Figure 4A). At 42 h, no significant difference was observed between the control group (2% FBS without Mitomycin C) and the one treated with 2 µg/mL Mitomycin C (Figure 4A). In contrast, 4 and 6 µg/mL Mitomycin C caused pronounced cytotoxicity, with most cells detached and floating, thereby preventing measurement (Figure 3A). Trypan Blue staining at the end of the treatment confirmed that 4 and 6 µg/mL Mitomycin C were highly cytotoxic, whereas 2 µg/mL effectively blocked cell division without causing significant toxicity (Figure 4B). Although quantitative wound closure analysis showed no difference between 2 µg/mL Mitomycin C and the control group, qualitative observations reflected the proliferation block induced by 2 µg/mL Mitomycin C. In the control group, wound areas exhibited a higher cell density with numerous rounded cells, indicating that closure involved both proliferation and migration (Figure 3B). By contrast, fewer and more elongated cells were observed in samples treated with 2 µg/mL Mitomycin C, suggesting that closure was primarily driven by migration (Figure 3B). Overall, continuous incubation with 2 µg/mL Mitomycin C for up to 42 h effectively inhibits proliferation without affecting viability or migration, making this condition suitable for wound healing assays of at least 42 h duration.

**Figure 5:**
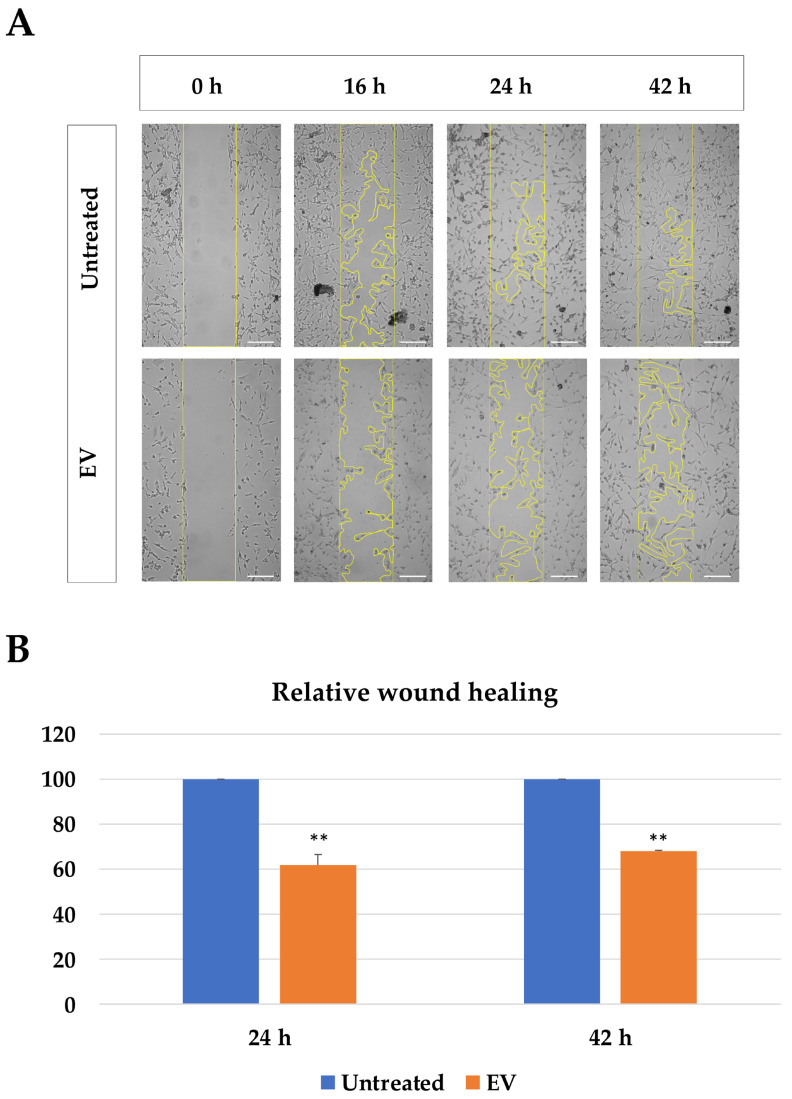
Impact of extracellular vesicles on wound healing in U87 glioblastoma cells. (**A**) Representative images of untreated and extracellular vesicle (EV)-treated U87 cells were acquired at 0, 16, 24, and 42 h after treatment. The thick yellow line indicates the cell-free area at each time point, while the thinner yellow outline shown at 24 and 42 h represents the original wound area at 0 h. Scale bar: 250 µm. (**B**) Quantification of wound closure over time. Wound healing is expressed as a percentage relative to untreated cells at the same time points. The data at 24 h represent the mean ± standard error of the mean (SEM) from four independent experiments (using three individual EV preparations and one EV reference pool), each performed in triplicate. At 42 h, the data represent the mean ± SEM from two of the four independent experiments (performed in triplicate), specifically based on EV Batches 1 and 3, as reported in Table 1. Statistical analysis was performed using a *t*-test; *p* < 0.01 is indicated by double asterisks (**).

The assay was used to evaluate the effect of NTERA2 EV samples on the migratory ability of GB cells, compared to untreated cells, by measuring the percentage of wound closure over time. Representative images captured at different times for both control (untreated) samples and the samples incubated with EVs, along with relative data analysis, are reported in Figure 5 and Table 3.

Figure 5 shows the quantification of wound closure over time. At 24 h, the data represent three individual EV preparations and one reference EV pool. At 42 h, the data refer to one of the individual EV preparations and the reference EV pool (Batches 1 and 3, respectively, in Table 1), for which both protein and nanoparticle concentration were determined. Using individual EV preparations enables precise assessment of batch-specific properties, whereas pooling multiple EV preparations provides a more representative sample of the overall population. This approach combines the consistency of pooled EVs with the specificity of individual batches, thereby enhancing the reliability of the experiments. All EV treatments were normalized based on protein concentration (10 µg/mL), with nanoparticle measurements performed for selected batches (Table 1). To standardize the protocol up to 42 h, Batch 1 (single preparation) and Batch 3 (pooled preparation) were selected based on their characteristics: (i) particle-to-protein ratio diversity: Batch 1 exhibited the lowest particle-to-µg ratio (1.44 × 10^8^ ± 3.20 × 10^7^), whereas Batch 3 showed the highest ratio (2.29 × 10^8^ ± 2.76 × 10^7^), allowing a broader range of particle concentrations to be tested; (ii) reduction in experimental variability: Batch 2 had a higher relative standard deviation (37%) compared to Batch 1 (22%) and Batch 3 (12%), indicating greater uncertainty in particle concentration per µg. Overall, results at both 24 and 42 h (Figure 5B) show a statistically significant reduction in cell migration in the presence of EVs (62.1% at 24 h, *p* = 0.0023 and 68.9% at 42 h, *p* = 0.0042) compared with untreated cells (100.0%).

## 5. Recommendations and Operational Notes

The protocol has been optimized to ensure reliable and consistent results, thereby avoiding the waste of time and money due to low reproducibility between technical and/or biological replicates. Table 4 provides a list of key process parameters, associated checkpoints, and preventive and corrective actions.

## 6. Discussion

The identification and dissemination of practical guidelines, protocols, and SOPs are essential to ensure that research is conducted in a reproducible, transparent, and accessible manner [56,57]. All variables that significantly influence the final results should be identified and controlled during the experiment, and the risk of failure should be minimized. Although the wound healing assay is seemingly straightforward to perform and analyze, it can still be inherently heterogeneous, potentially concealing complex underlying dynamics. Here we summarize the key points that characterize our protocol, which collectively improve reproducibility and quality control:

(1) We used a cell-exclusion approach based on the use of ibidi silicone culture inserts to create a physical barrier and generate the wound. Cells are seeded into the culture inserts to leave a reproducibly sized cell-free gap when the inserts are removed. This approach has the important advantage of creating more homogeneous and reproducible gaps, without damaging the cells or activating a cellular program that could interfere with the measurement of cell migration. In the most commonly used cell depletion approach, instead, cells are usually seeded into 6- or 12-well plates, and then the cell monolayer is scratched with a P20 yellow tip to create a wound (scratch assay). Despite its simplicity, time and cost effectiveness, the scratch assay does have important limitations. The main weakness lies in the low reproducibility of the scratch, which can result in the wound areas being very heterogeneous, thereby affecting both reliability and reproducibility of the results. Moreover, the scratch may cause damage to cells at the edge of the wound, which could affect cell migration and introduce additional variability to the assay. The smaller size of the inserts compared to 6- or 12-well plates normally used for making the scratch, offers the significant advantage of reducing the amounts of cells, medium, reagents and especially of EVs needed for the assay. Last, but not least, the reduced insert size and well-defined outer edge of the insert facilitate spatial referencing, particularly under manual, non-motorized acquisition settings. However, the smaller surface area also requires extreme care and precision when setting up the experiments, as detailed in the protocol;
(2) We set up continuous incubation with an antimitotic drug such as Mitomycin C to inhibit cell proliferation for 42 h, avoiding confounding effects on the evaluation of cell migration. We used the drug for all the length of the treatment, instead of the shorter preincubation period usually employed [58,59]. This prevents partial and uncontrolled inhibition of cell proliferation, especially in treatments longer than 24 h, which could distort the determination of the effects of EV treatment specifically on cell migration. The concentration of Mitomycin C was carefully determined to limit cell proliferation while avoiding toxic effects on the cells, enabling the test to be performed for 42 h. A concentration of 2 μg/mL has been confirmed across different batches of Mitomycin C and GB U87 cells. However, we recommend preliminary dose testing with different Mitomycin C types or batches and/or different cell lines.
(3) We set up the GB cell number (18,000 cells/insert) to seed in the presence of an antimitotic treatment (Mitomycin C) to create a defined but not-overgrown cell monolayer, suitable for assays up to 42 h, without changing the Culture Medium. This set up was performed with U87 cells, but could serve as a reference point to other GB or tumor cells.
(4) U87 GB cells are characterized by a highly adhesive phenotype, making them especially sensitive to minor variations during the seeding procedure. As a consequence, small differences in the initial cell distribution within the insert, potentially arising from microflows during the addition of the cell suspension and/or movements of the well plate during adhesion, can be amplified over time. Due to the reduced surface area of the insert, these early asymmetries may become increasingly evident during wound closure, particularly under treatment conditions, and should therefore be carefully considered when interpreting migration data. This is particularly true when cells do not migrate as a compact front but rather independently, as in the case with U87 cells, meaning that the distance between the two fronts can be extremely heterogeneous. Taking two images of each technical replicate at 5× magnification and measuring the entire gap area corresponding to the sum of the two imaging fields can provide an accurate representation of cell migratory behavior inside the well.
(5) We established a reference framework for EV samples to be tested in the U87 GB wound healing assay, taking into account protein concentration, particle size and concentration, and particle-to-protein ratio. In particular, we provide a valuable reference for 10,000× *g* sedimented EV fractions, which remain less extensively characterized than 118,000× *g* isolated EVs, despite attracting increasing interest. The particle-to-protein ratio has been proposed as an indicator for estimating the purity of EV preparations [60], although its applicability requires careful evaluation [49,50], since expected ratios can vary depending on multiple factors, such as EV origin and isolation method. Notably, the particle-to-protein ratio of NTERA2-derived EV preparations is consistent with values reported for large EV fractions of similar size, isolated from other cancer cell lines (e.g., breast) either after 16,500× *g* sedimentation alone or followed by density cushioning [53].
(6) Unless otherwise specified, we suggest beginning testing with a protein concentration of 10 µg/mL. For NTERA2 EV batches, this corresponds to an estimated assay concentration of 1.92 × 10^9^ ± 4.35 × 10^8^ particles/mL. This value should be considered an approximate average, since not all of the preparations included in this protocol have been individually quantified. For dose–response evaluation, we suggest beginning with EV concentrations within the range of 5–20 µg/mL of protein, which for our EV preparations corresponds to approximately (9.60 ± 2.18) × 10^8^ to (3.84 ± 0.87) × 10^9^ particles/mL. Concentrations below this range may be insufficient to produce a reproducible and stable effect, while concentrations above this range may lead to EV aggregation, which can affect EV uptake and subsequent phenotypic responses. Different EV subtypes and fractions may require specific dose optimization due to heterogeneity in size, particle-to-protein ratio, physicochemical properties, molecular content, and functional activity, as well as in isolation and characterization methods. Nevertheless, our reference provides a valuable benchmark for setting up experiments, as well as for analyzing and comparing results across EVs from different sources.

## 7. Conclusions

The described protocol was successfully applied to study the impact of NTERA2-derived EVs on the migration of U87 GB cells. Although it was designed for a specific type of EVs and cells, it provides an important framework for reference and comparison in studies of EV-mediated cell migration. The protocol includes all the necessary details for full replication, as well as guidance on identifying and accounting for potential sources of experimental failure. Furthermore, it can be readily adapted to other GB cell lines and/or adherent tumor cell cultures, provided that critical process parameters are appropriately identified and described. Taken together, these methodological improvements offer a robust and quantitative platform for investigating EV effects on cell migration, representing a significant advancement over traditional wound healing assays.

## 8. Patents

G.L.L. and F.M. are inventors on the patent application WO/2023/199237, related to the use of Cripto-positive lipidic vesicles for the treatment of aggressive tumors [61]. The Italian patent IT202200007580 has been granted in 2024. The European patent application EP4507714 and the U.S. patent application US20250255983 are currently under examination.

## Figures and Tables

**Table 1:** Median particle diameter, particle and protein concentrations, and particle-to-protein ratio of NTERA2 extracellular vesicle batches. The first two batches of extracellular vesicles derive from two independent cell preparations, while the third batch is a pooled reference preparation (not including the first two batches). Batches 1 and 3 were used in the wound healing assay.

EV Batch	Median Diameter (nm)	Particle Concentration (Particle/mL)	Protein Concentration (μg/mL)	Particles/μg of Protein
1	184.67 ± 7.09	(6.90 ± 1.53) × 10^10^	480	(1.44 × 10^8^) ± (3.20 × 10^7^)
2	171.25 ± 10.24	(7.68 ± 2.82) × 10^10^	380	(2.02 × 10^8^) ± (7.41 × 10^7^)
3	172.67 ± 4.04	(1.93 × 10^11^) ± (2.33 × 10^10^)	843	(2.29 × 10^8^) ± (2.76 × 10^7^)

**Table 2:** Representative wound healing measurements at different Mitomycin C concentrations and time points. The images were acquired using Leica DFC 480 RGB camera and analyzed using ImageJ software (version 1.54g). Abbreviations: FBS, Fetal Bovine Serum; Mit C, Mitomycin C.

Treatment	*t*_0_	*t*_1_ (24 h)		*t*_2_ (42 h)	
	Wound Area (mm^2^)	Wound Area (mm^2^)	% Wound Healing ((*t*_0_ Area − *t*_1_ Area)/*t*_0_ Area) × 100	Wound Area (mm^2^)	% Wound Healing ((*t*_0_ Area − *t*_2_ Area)/*t*_0_ Area) × 100
**2%FBS**	1.126	0.376	66.6	0.000	100.0
**2%FBS** **2 μg/mL Mit C**	1.076	0.371	65.5	0.160	85.1
**2%FBS** **4 μg/mL Mit C**	1.039	0.540	48.0	-	-
**2%FBS** **6 μg/mL Mit C**	1.166	0.669	42.6	-	-

**Table 3:** Representative wound healing measurements of U87 untreated and extracellular vesicle-treated cells at different time points. The images were acquired with the Leica DFC 480 RGB camera and analyzed using ImageJ software (version 1.54g). Abbreviations: EV, extracellular vesicle.

		Untreated	EV
***t*_0_**	**Wound Area (mm^2^)**	1.219	1.122
***t*_1_** **(16 h)**	**Wound Area (mm^2^)**	0.384	0.843
	**Wound Healing (%)** **((*t*_0_ Area − *t*_1_ Area)/*t*_0_ Area) × 100**	68.5	24.9
***t*_2_** **(24 h)**	**Wound Area (mm^2^)**	0.207	0.698
	**Wound Healing (%)** **((*t*_0_ Area − *t*_2_ Area)/*t*_0_ Area) × 100**	83.0	37.8
***t*_3_** **(42 h)**	**Wound Area (mm^2^)**	0.146	0.502
	**Wound Healing (%)** **((*t*_0_ Area − *t*_3_ Area)/*t*_0_ Area) × 100**	88.0	55.3

**Table 4:** Critical process parameters and checkpoints.

Critical Step	Critical Process Parameter	Monitoring and Preventive/Correcting Action
*Day 1 Step 2*	Cell detachment	Incomplete or excessive detachment may affect cell viability and downstream outcomes. If detachment is excessive, reduce Trypsin incubation time and/or temperature; if insufficient, increase incubation time or use a different batch of Trypsin. Optimization may be required when adapting the protocol to different cell lines.
*Day 1 Step 9*	Insert adherence	Ensure that the silicone insert remains securely positioned throughout handling. Instability may lead to irregular wound formation.
*Day 1 Step 10*	Cell seeding density and distribution	To ensure the formation of a defined and homogeneous cell monolayer that does not overgrow after 24 h, both seeding density and homogeneity of cell distribution have to be monitored. Increased cell density might cause cellular stress and alter cell features, especially in a 42 h-long assay. To avoid this, accurate cell counting is essential. U87 cells are highly adhesive. Furthermore, due to the small size of the insert, even minor differences in the initial cell distribution can be markedly amplified during the experiment or upon treatment. To achieve a more uniform cell distribution, pipette slowly, and dispense the cell suspension against the wall of the insert. Allow the cells to settle undisturbed for 10–15 min.
*Day 2 Step 1*	Cell confluence and distribution at *t*_0_	Before removing the insert, check under the microscope that the cells have reached the desired level of confluence. If necessary, extend the incubation time until the desired confluence is achieved. Check that the U87 cell layer is homogeneous along all the wound borders. If significant differences in cell density or distribution are present at *t*_0_, consider discarding the corresponding wells. For other cell lines, check the required growth conditions and medium, and determine the adequate cell seeding density.
*Day 2 Step 2*	Insert removal and wound formation	Check that all wounds at *t*_0_ are regular and approximately 500 µm wide, as would be expected when using ibidi culture inserts. Exclude any wells displaying evident mechanical artifacts or incomplete gap formation. To select the most suitable ones for the assay, it is recommended to prepare more wounds than required.
*Day 2 Step 3*	Concentration of serum and antimitotic drug	Check that cell density does not increase during the experiment, as this would indicate ongoing proliferation which would contribute to wound closure and potentially confound the results. If this occurs, repeat the experiment and verify that the experimental conditions (e.g., serum levels and/or antimitotic drug concentrations) are appropriate. Also, check that the antimitotic drug is not past its expiry date and is being stored properly.
*Day 2 Step 4 and 6*	Selection and tracking of imaging areas	Imaging areas should be consistently defined and re-identified across time points using either motorized stage coordinates or manual reference landmarks, to ensure comparability between acquisitions. Two non-overlapping fields per well are recommended to capture the wound area over time.
*Day 2 Step 6*	Time points	Adjust the time points according to the speed of cell migration, preferably within a 42 h window. Do not exceed 48 h to avoid changes in culture pH, nutrient depletion, or prolonged exposure to Mitomycin C, which could affect cell viability and behavior. If necessary, replace the medium or treatment during the assay.
*Day 3 Step 1*	Calculation of the wound area	Irregular wounds may affect the reproducibility of the assay and confound the effect of the EV treatment compared to untreated cells. Discard cell samples in which the *t*_0_ area differs significantly from the expected size. This could be due to the ibidi inserts not adhering properly (cells invade the area under the insert, resulting in a smaller wound), cells detaching when the inserts are removed or low cell density (resulting in a larger wound).
*Statistical Analysis*	Standard deviation among biological replicates	Check the standard deviation or standard error of the mean across biological replicates. Assess the quality of cell culture batches (e.g., Mycoplasma absence, cell passage, cell viability, and metabolic activity) as well as EV preparations (e.g., nanoparticle size distribution, concentration, and purity), discarding any batches that show issues. If necessary, repeat the experiment to ensure reliable results.

## Data Availability

The data supporting the conclusions of this article will be made available by the corresponding author on request.

## Abbreviations

The following abbreviations are used in this manuscript:

BCA	Bicinchoninic Acid
CD63	Cluster of Differentiation 63
CO_2_	Carbon Dioxide
D	Diameter
DMEM	Dulbecco’s Modified Eagle’s Medium
EVs	Extracellular Vesicles
FBS	Fetal Bovine Serum
g	Gravity
GB	Glioblastoma
H	Height
HSP70	Heat Shock Protein 70
MISEV	Minimal Information for Studies of Extracellular Vesicles
N	Number
PBS	Phosphate-Buffered Saline
PCR	Polymerase Chain Reaction
PFA	Paraformaldehyde
px	Pixels
t	Time
V	Volume
WA	Wound Area
WH	Wound Healing
